# Burnout in the Emergency Department: Survey of Prevalence and Modifiable Risk Factors

**DOI:** 10.5811/westjem.24872

**Published:** 2025-09-25

**Authors:** Matthew Kraus, Michelle Fischer

**Affiliations:** * Penn State College of Medicine, Hershey, Pennsylvania; † Penn State College of Medicine, Department of Emergency Medicine, Hershey, Pennsylvania

## Abstract

**Introduction:**

We assessed the prevalence of burnout syndrome among emergency physicians and advanced practice practitioners (APP) in an academic emergency department (ED) to identify demographic and lifestyle factors associated with burnout.

**Methods:**

We administered a cross-sectional survey including the Maslach Burnout Inventory (MBI) with a demographic/lifestyle component to emergency physicians, residents, and APPs at an academic ED. We reported descriptive data and performed chi-square analysis to identify significant variables, followed by logistic regression to quantify their effects. A factor count was performed to assess for additive effects of burnout risk factors.

**Results:**

We collected 55 surveys (60% response rate) yielding an overall burnout prevalence of 52.7%. The following had a significant association with burnout: 0–6 days off per month; fewer than two major hobbies; thoughts of quitting one’s job “at least some of the time”; and spending less than four hours outdoors per week. Zero to six days off per month was associated with 4.70 times more burnout compared to ≥7 days off per month (95% confidence interval [CI] 1.24–17.82). Participants who met 3–4 vs 0–2 of the previously mentioned conditions had a 6.87 times increased burnout prevalence (95% CI 2.01–23.52).

**Conclusion:**

This preliminary study highlights four unique factors associated with burnout. It also demonstrates that a specific number of days off may reduce burnout prevalence. Emergency department wellness efforts should consider focusing on strategically scheduling time off each month while encouraging individual habit generation and time spent outdoors to maximize burnout protection. Further research is needed to evaluate the efficacy of the proposed interventions.

## INTRODUCTION

The Maslach Burnout Inventory (MBI) defines burnout as a syndrome composed of three types of feelings: emotional exhaustion (EE); depersonalization (DP); and low sense of personal accomplishment (PA).^1^ Emotional exhaustion refers to feelings of mental fatigue and overextension from one’s work. Depersonalization refers to an unfeeling and impersonal response to recipients of one’s care. Personal accomplishment refers to feelings of competence and success within one’s area of work.^1^ The prevalence of burnout among US physicians is elevated compared to the general US population (37.9 vs 27.8%).^2^ Burnout remains an ongoing area of concern as its effects are significant for physicians and their patients.^3^,^4^

Two concerning patient consequences, increased susceptibility to medical errors and lower quality care, have direct effects on patient safety and satisfaction.^3^ Consequences of physician burnout include depression, substance use disorder, poor habit generation, and suicide.^3^,^4^ Burnout has negative effects on the entire medical system as it leads to increased physician turnover and reduced productivity.^3^ Emergency medicine (EM) consistently ranks among the highest in reported rates of burnout among medical specialties^4^,^5^,^6^,^7^ and has the highest burnout rate (65%) according to a recent Medscape Physician Lifestyle and Happiness Report.^6^ (Note that this report’s discussion reflects the presumed impact of the COVID-19 pandemic on these results.)

Unlike most specialties where hours worked directly correlate with increased burnout, emergency physicians experiences high burnout despite working fewer overall hours than those in many other specialities.^4^ This phenomenon may be partially explained by stressors that are unique to EM such as caring for multiple high-acuity patients simultaneously, frequent task-switching, patient and colleague rudeness, litigation stress, sleep-schedule switching, and being faced with constant uncertainty.^4^ Multiple studies have shown that factors associated with burnout include younger age or junior status, number and duration of night shifts, increased workload, acuity of care, decreased resilience, and a desire to quit one’s current job.^5^,^7^,^8^,^9^ There is less existing research focused on the additive effect of multiple burnout risk factors. Furthermore, prior studies have not shown a specific number of days off that markedly reduces burnout prevalence.

Our goal in this study was to identify the prevalence of burnout among attending physicians, residents, and advanced practice practitioners (APP) within a single, large, academic emergency department (ED). We sought to compare rates of burnout stratified by level of training and compare them to prior studies while correlating rates of burnout with lifestyle factors to identify interventions for burnout reduction.

## METHODS

We employed a cross-sectional study design using a validated survey tool, the 22-item Maslach Burnout Inventory Human Services Survey for Medical Professionals (MBI-HSS-MP) and a paired 24-item demographic and lifestyle factor survey that was created for this research. The study was submitted to an institutional review board and was deemed exempt due to lack of inclusion of identifying information. The survey was performed between September 2022–January 2023 within the ED at a single, large, academic medical center with a volume of over 80,000 visits per year and a three-year, Accreditation Council for Graduate Medical Education (ACGME)-accredited residency program in the northeastern United States.

The survey was offered voluntarily to approximately 90 attending physicians, residents, and APPs within the ED. The primary outcomes were scores in the three domains of the MBI. A yes/no categorization of burnout presence was completed by defining burnout as a high-range score in either the EE or DP domains of the MBI. Although no formal method of characterizing the presence or lack of burnout exists according to the MBI, many prior studies using the MBI share this unofficial definition. The other variables measured by the survey included demographic and lifestyle factors. A full copy of the survey is available upon request. We collected and managed survey data using Research Electronic Data Capture (REDCap)^10^ tools hosted at Penn State Hershey Medical Center. The survey was disseminated via email and QR codes posted in the ED. Emails were sent twice to all potential participants, and an announcement was made at a resident conference, attending physician meeting, and APP meeting to increase participation.

**Population Health Research Capsule**

What do we already know about this issue?

*Burnout is more prevalent among physicians than the general population and more prevalent among emergency physicians compared to other medical specialties*.

What was the research question?

*What demographic and lifestyle factors in isolation or combination are associated with burnout?*

What was the major finding of the study?

*Having 3–4 vs 0–2 of the identified risk factors increased burnout prevalence by 6.87 times (P=.0013, 95% CI 2.01–23.52)*.

How does this improve population health?

*Recognizing key drivers of burnout among emergency physicians is essential to tailoring appropriate interventions to prevent and remedy burnout*.

### Statistical Analysis

We chose to group responses for continuous variables such as weekly hours worked, salary, etc, by combining them into five ranges of values equally spread across a range of values estimated to include most survey responses. To analyze the association of these variables with burnout, we analyzed burnout prevalence on either side of cutoffs marked by these ranges. Statistical analysis was performed using SAS v9.4 (SAS Institute Inc, Cary, NC).

We reported descriptive statistics for burnout prevalence and performed chi-square analysis to assess for association of demographic or lifestyle variables with burnout prevalence using relative risk. A multivariate binary logistic regression model was developed using the four variables with *P*-values <.05 in the univariate chi-square analysis. Given 55 study subjects, the rule of 10 supported inclusion of four variables in the model. To assess for strength of the model, we generated a receiver operator curve (ROC) and calculated the area under the curve (AUC). For the final model we calculated sensitivity, specificity, likelihood ratios, positive predictive value, and negative predictive value. A chi-square analysis with odds ratio calculation was completed to assess for an association between the number of burnout covariates an individual reported and the prevalence of experiencing burnout.

## RESULTS

A total of 55 survey results were collected from 26 residents, 20 attending physicians, and 9 APPs for an overall response rate of approximately 60%. The demographics of the respondents are reported in Table 1. Of the individuals surveyed, 52.7% met the criteria for burnout. The mean scores and standard deviations of the whole cohort for EE, DP, and PA were 25.7 ± 11.2, 10.5 ± 6.1, and 34.6 ± 8.0, respectively. Table 1 displays the overall burnout prevalence and the prevalence across the three burnout domains. Higher EE and DP and lower PA scores indicate a higher degree of burnout. The burnout rates among residents, attending physicians, and APPs were similar at 50.0%, 55.0%, and 55.6%, respectively (*P*=.93). Residents yielded the highest DP scores (11.6 ± 7.8) while APPs had the highest EE scores (27.1 ± 7.3) and lowest PA scores (32.2 ± 5.3); however, these differences were not statistically significant (*P*=.46, *P*=.42, *P*=.29). Chi-square univariate analysis for possible burnout predictors showed four variables with a significant relationship to burnout prevalence (Table 2): having 0–6 days off per month (relative risk [RR] (relative risk) 1.94, 95% confidence interval [CI] 1.01–3.73), less than two major hobbies (RR 1.71, 95% CI 1.04–2.82), thoughts of quitting one’s job “at least some of the time” (RR 1.71, 95% CI 1.04–2.82), and spending fewer than four hours outdoors per week (RR 1.80, 95% CI 0.94–3.44). Although the CI for time outdoors per week crosses one, the *P*-value was noted to be significant at *P*=.0466 and, therefore, we included the variable in the multivariate logistic regression model. We created a multivariate binary logistic regression model to further assess the effect of these four variables on burnout (Table 3). When controlling for hobbies, time spent outdoors, and thoughts of quitting, number of days off per month was found to be a significant predictor of burnout (adjusted OR 4.70, 95% CI 1.24–17.82). Greater than seven days off per month did not confer additional reduction in burnout prevalence. Other predictors did not have significant contribution to the model when controlled for other predictors. A ROC demonstrated a fair-to-good performance of the model (AUC 0.794, *P*<.0001, 95% CI 0.676–0.913) (Figure).

The model demonstrated a sensitivity of 79.3%, specificity of 61.5%, positive likelihood ratio of 2.06, negative likelihood ratio of 0.34, positive predictive value of 69.7%, and negative predictive value of 72.7%. The likelihood ratios indicate fair efficacy of the model in adjusting pre-test probability. If using 0–6 days off per month, < two major hobbies, thoughts of quitting “at least some of the time,” and < four hours spent outdoors per week as risk factors for burnout, chi-square analysis indicates that having 3–4 vs 0–2 of these conditions increased burnout prevalence by 6.87 times although the CI was notably wide (95% CI 2.01–23.52). This indicates that possessing more risk factors for burnout increases the likelihood that they will screen positive for burnout by the MBI.

## DISCUSSION

This study demonstrated a burnout rate of 52.7% among emergency attending physicians, residents, and APPs at a single, large, academic ED, which is comparable to rates reported in prior studies.^11^,^12^,^13^,^14^ In a study of French ED staff conducted by Moukarzel et al, 50.7% of emergency physicians met criteria for burnout.^11^ This was based on the definition of burnout as a high-range score in EE or DP, which has been used in several other studies but remains without clear consensus.^11^ A review article by Arora et al found emergency physician burnout rates to range between 60–65% using the MBI.^12^ Another review by Zhang et al showed that among 1,255 emergency physicians, 40% and 41% possessed high scores in EE and DP, respectively.^13^

Takeyesu et al performed a study among EM resident physicians and detected a burnout rate of 65%.^14^ They also determined that having a spouse or significant other, poor global job satisfaction, lack of administrative autonomy, lack of clinical autonomy, and intolerance of uncertainty were associated with increased burnout.^14^ We found no significant difference in the overall burnout rate or the various burnout domains (EE, DP, and PA) among the subpopulations of attending physicians, residents, and APPs. There was a trend toward residents displaying the highest depersonalization levels while APPs displayed the highest emotional exhaustion and lowest personal accomplishment levels, but these differences were not statistically significant.

This study identified four unique factors that showed an association with burnout: 0–6 days off per month, having < two major hobbies, having thoughts of quitting, and spending < four hours outdoors per week. Days off per month was the most significant variable contributing to burnout prevalence in our multivariate model. Prior studies have associated emergency physician burnout with a desire to quit one’s job, junior- learner status, and increased shift number and duration,^7^,^11^ which is partially congruent with our results. There appeared to be an additive effect on burnout prevalence when an individual reported more than one of these factors. In our study, an individual having 3–4 of the factors had seven times the risk of burnout when compared to having ≤2. Additional studies are required to validate these variables and assess their additive effects.

Burnout is highly prevalent in EM. Prior studies suggest that the most useful burnout inventories are completed on the departmental level to create interventions based on that department’s unique burnout profile. Examples of different burnout profiles include the disengaged type (high DP), overextended type (high EE), and ineffective type (low PA).^15^ This profile would be reflected by targeted feedback from members of the department’s workforce or collection of specific MBI survey data. In a recent article, Guille et al conclude that due to variation in measurements of burnout, assessment of depression may be a more fitting method of assessing healthcare workers’ well-being, which was supported by further correspondence with Bianchi.^16^,^17^

While many risk factors may be universal to EM, department-specific factors may be easier to intervene upon. Montgomery et al suggested that optimizing the relationship between institutions and employees by empowering employees to have increased control over their work environment is helpful in reducing burnout.^15^ This is consistent with other literature related to burnout, which frequently suggests that burnout is inversely related to an individual’s sense of personal control or autonomy. This concept is highlighted by the findings of Takayesu et al, which associated EM resident physicians’ lack of administrative and clinical autonomy with increased burnout.^14^ There is evidence to suggest that systems-based interventions are far superior to suggestions of individual behavior modification when striving to reducing the rate of burnout.^18^ Additionally, departmental efforts focused on encouraging increased time spent outdoors and hobby generation may have direct effects on decreasing healthcare burnout.

Our study suggests that creating clinical schedules with at least seven days off per month may minimize burnout in EDs and should be implemented whenever possible. Regarding resident schedules, it may be necessary to consider a modification to the current ACGME work-hour rules to minimize burnout. Building in a minimum number of seven days per month with no scheduled clinical or didactic work could be a beneficial change from the current 24-hour free period that is required on a weekly basis. To accomplish this, EM programs may be required to assign more residents to ED-specific rotations simultaneously to fulfill necessary coverage of shifts. While such an intervention may be beneficial to EM residents, we recognize that its implementation would be challenging and that other factors must be optimized to improve the status of burnout in the ED. Guille et al highlight that in addition to high volumes of work hours, the actual workload and documentation responsibilities are key contributors to burnout.^16^ Individual departments and residency programs should focus on tailoring interventions to the specific feedback of their workforce in a way that prioritizes employee autonomy, multidimensional wellness, and specific elements of employee burnout profiles if collected.

## LIMITATIONS

This study’s cross-sectional survey nature is an inherent limitation as it could not demonstrate causality. This study was also limited by a self-selection bias due to voluntary participation. The single-center nature of this study may decrease external validity, particularly for EDs with volume and structure that is different from our institution. “Number of days off per month” were initially recorded in grouped ranges in our survey and may have biased respondents into choosing a middle value such as “4–6” without explicitly comparing to their true schedules. This study was also performed with a small sample size and was likely underpowered to detect additional significant associations of lifestyle factors with burnout prevalence. Additional risk factors associated with burnout may have been discovered with increased sample size and power.

## Figures and Tables

**Figure:**
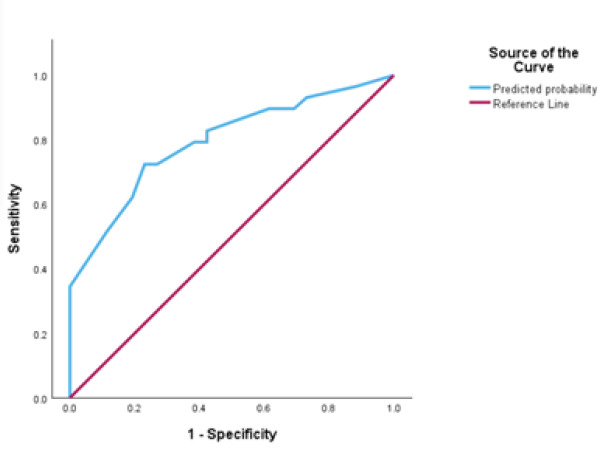
Receiver operator curve for multivariate model performance in a study on physician burnout in the emergency department. *ROC*, receiver operator curve.

**Table 1:** Study demographics and burnout prevalence.

Variable	Characteristics	Percentage (N=55)	Fraction with Burnout	% with Burnout
Sex	Male	60.0	16/33	48.5
	Female	40.0	13/22	59.1
Level of training	Attending	36.4	11/20	55.0
	Resident	47.3	13/26	50.0
	APP	16.4	5/9	55.6
Marital status	Married	63.6	20/35	57.1
	Single	36.4	9/20	45.0
Employment type	Full-time	89.1	24/49	49.0
	Part-time	10.9	5/6	83.3
Population served	Adult and pediatric	74.6	22/41	53.7
	Adult only	12.7	4/7	57.1
	Pediatric only	12.7	3/7	42.9
Presence of burnout	Frequency (N=55)			
Yes	29	52.7		
No	26	47.3		
Burnout domain	Score range			
Emotional exhaustion	Low (0–18)	27.3		
	Moderate (19–26)	27.3		
	High (≥27)	45.5		
Depersonalization	Low (0–5)	21.8		
	Moderate (6–9)	27.3		
	High (≥10)	50.9		
Personal achievement	Low (≤40)	34.6		
	Moderate (34–39)	25.5		
	High (0–33)	40.0		

*APP*, advanced practice practitioner.

**Table 2:** Association of burnout with demographics and lifestyle factors univariate analysis.

Variable	% with Burnout	Chi-square P-value*	Relative Risk	RR 95% CI	Unadjusted Odds Ratio	OR 95% CI
Gender		.44	0.82	0.50–1.35	0.65	0.22–1.94
Male	48.5					
Female	59.1					
Marital Status		.39	0.79	0.45–1.38	0.55	0.18–1.69
Single	45.0					
Married	57.1					
Children		.85	0.95	0.58–1.57	0.90	0.31–2.63
0	51.6					
1+	54.2					
Employment		**	0.59	0.37–0.93	0.19	0.02–1.77
Full-time	49.0					
Part-time	83.3					
Setting		.81	0.93	0.51–1.69	0.86	0.26–2.91
Adult or pediatric	50.0					
Adult and pediatric	53.7					
Hours worked/week		.55	0.85	0.50–1.46	0.72	0.24–2.14
30–49	47.6					
50+	55.9					
Overnight shifts/month		.72	0.91	0.55–1.52	0.82	0.28–2.40
0–3	50.0					
4+	54.8					
Days off/month		.02	1.94	1.01–3.73	3.67	1.16–11.56
0–6	64.7					
7+	33.3					
Leave of absence		**	0.78	0.34–1.81	0.54	0.05–6.33
Never	51.9					
Once	66.7					
Salary		.60	0.88	0.53–1.44	0.75	0.25–2.24
0–150k	50.0					
151k+	57.1					
Tobacco use		**	0.77	0.41–1.44	0.52	0.09–3.11
Never	51.0					
Any use	66.7					
Practice instrument		**	1.28	0.55–2.98	1.85	0.16–21.70
Yes	66.7					
No	51.9					
Major hobbies		.03	1.71	1.04–2.82	3.34	1.07–10.39
0–1	69.6					
2+	40.6					
Hours of sleep/day		.50	1.19	0.72–1.97	1.44	0.50–4.16
5–6	57.1					
7–8	48.1					
Thoughts of quitting		.03	1.71	1.04–2.82	3.34	1.07–10.39
Sometimes/often	69.6					
Never/rarely	40.6					
Time outdoors/week		.05	1.80	0.94–3.44	3.14	1.00–9.89
0–3	62.9					
4+	35.0					
Caffeinated beverages/day		.17	1.42	0.88–2.31	2.21	0.71–6.86
0–1	65.0					
2+	45.7					
Perceived institutional support		.15	1.57	0.79–3.12	2.40	0.72–7.93
Unsupported/Mild	59.0					
Well-supported	37.5					
Alcoholic beverages/week		.79	1.07	0.64–1.78	1.15	0.38–3.48
0	55.0					
1+	51.4					
Perceived social support		**	1.15	0.54–2.48	1.38	0.21–9.01
Unsupported/Mild	60.0					
Well-supported	52.0					
Family proximity		.82	0.93	0.50–1.76	0.87	0.24–3.13
In-home	50.0					
In-state/Out-of-state	53.5					

* *P*-values are considered significant if <.05 to warrant inclusion in multivariate model.

** Chi-square *P*-value not calculated due to expected value <5.

*RR*, relative risk; *CI*, confidence interval; *OR*, odds ratio.

**Table 3:** Multivariate logistic regression model.

Effect	Chi-square P-value	Adjusted odds ratio	OR 95% CI
0–6 vs. 7+ days off/month	.02	4.70	1.24–17.82
Thoughts of quitting vs. never/rare thoughts of quitting	.06	3.45	0.96–12.35
0–1 vs. 2+ hobbies	.16	2.68	0.67–10.80
0–3 vs. 4+ hours outdoors/week	.35	1.92	0.49–7.54

*OR*, odds ratio; *CI*, confidence interval.
